# Opportunities for public involvement in big data research in palliative and end-of-life care

**DOI:** 10.1177/02692163211002101

**Published:** 2021-03-24

**Authors:** Halle Johnson, Joanna M Davies, Javiera Leniz, Emeka Chukwusa, Sarah Markham, Katherine E Sleeman

**Affiliations:** 1 Cicely Saunders Institute, King’s College London, London, UK; 2 Patient and Public Involvement Contributor, Cicely Saunders Institute, King’s College London, London, UK; 3 Visiting Researcher, Department of Biostatistics and Health Informatics, King’s College London, London, UK

Public involvement – the process by which research is conducted in collaboration with patients, carers or members of the public – is increasingly considered a prerequisite for high-quality research.^1^ Evidence suggests that public involvement can benefit research by helping to identify and prioritise patient-focused research questions, aiding recruitment and retention, and helping to foster greater links between researchers and the wider community.^2–4^

Public involvement is encouraged across the research cycle and guidance has been developed to support researchers to involve the public at each stage.^5,6^ However, currently, such guidance is focused on studies which include primary data collection, and there is little guidance on how to meaningfully involve the public in big data research.

Big data research takes many forms.^7,8^ In palliative care, this research often involves secondary analysis of routinely collected data (i.e. data initially collected for other purposes other than research, as part of a standard administrative or care process) such as death registry data and electronic health records.^9,10^

Differences in the reported presence of public involvement across studies have shown that involvement in big data research is significantly limited in comparison with other study designs.^11^ It has been suggested that this may be because common approaches to public involvement adopted in primary data research are not appropriate within big data analysis studies.^12,13^ While public members are commonly involved in primary research to review and develop patient information leaflets or other research materials, undertake interviews with research participants or even support recruitment for a clinical trial, these involvement opportunities are not applicable to big data research.^13,14^ In addition, the highly data driven discussions that underline this type of research can present a barrier to public involvement with ‘unfamiliar’ and ‘scientific’ language repeatedly being cited as an obstacle, across fields.^15^ Due to this there is now growing recognition that public involvement in big data research requires special considerations.^13^

With the increasing opportunity for big data research in palliative and end-of-life care,^9^ and in parallel, a requirement by many funders, both nationally and internationally, to actively involve the public within research, we aimed to explore both the challenges and opportunities for public involvement in big data research to understand how best to involve the public in these studies.

In November 2019, we held a full-day public involvement workshop at the Cicely Saunders Institute of Palliative Care and Rehabilitation at King’s College London on the theme ‘large dataset research in palliative care’. The purpose of the workshop was to explore public views on involvement within big data research. The workshop was attended by 10 patient, carer and public representatives with lived experience of, or interest in, palliative and end of life care and seven researchers. During the workshop public attendees were introduced to key concepts of big data research, initiating group discussion around data availability and access, the advantages and disadvantages of this kind of research, and data governance and ethics. In the afternoon, two researchers led a discussion to understand public views on the perceived challenges, opportunities, and value of involvement in these studies.

While most public attendees had experience of involvement in palliative care research, few had experience in big data research and perceived that over-use of jargon (e.g. ‘routine data’) was an initial barrier to involvement in this research. Many were also unaware of the concept of ‘big data’; what kinds of data were available and how data could be used for research. Acknowledging these challenges, public attendees felt their involvement in these studies remained important. The discussion led to several perceived potential opportunities for public involvement in big data research (see Table 1).

**Table 1.** Opportunities for involving the public in big data research in palliative and end of life care.

Raise public awareness of big data research • Public awareness of big data research projects should be raised, including increasing understanding of terminology such as ‘routinely collected’, ‘big data’ and ‘secondary analysis’, how data are accessed for research, what data are provided to researchers (e.g. specific variables), how data are provided (e.g. anonymised, safely transferred), how patient confidentiality is maintained, and the benefits and limitations of the use of these types of data in research. Involve patients, carers and the public in research priority setting with existing data sets • Palliative care researchers and/or research groups should develop a list of palliative and end of life care research questions which could be answered using currently available data sets and work with patients, carers and the public to prioritise which research questions to pursue. Involve patients, carers and the public to ‘humanise’ big data • Patients, carers and the public should be involved in the interpretation of the results, particularly in providing personal narratives to compliment or rebut trends and patterns in the data. Further work is needed to understand the best ways of involving patients and the public at this stage to ensure involvement is meaningful. Prioritising next steps for research • Patients, carers and the public could help develop and advise on future research questions arising from trends and patterns identified in the data. Follow-up qualitative studies, which patient and public involvement contributors could continue to be involved in, were perceived to be one way to ensure continued and meaningful involvement. Involve patients, carers and the public in the wider lifecycle of big data • Patients, carers and the public felt they should play a role in helping to push the agenda of standardising and collecting data from relevant palliative and end of life care services such as care homes and hospices, and also help researchers to develop person-centred palliative and end of life care outcomes which would lend themselves to routine collection. • Patients, carers and the public should be involved in the governance and curation of national and local data sets to ensure patient confidentiality is maintained.

Public involvement could enhance the relevance and impact of big data research in palliative care, by helping researchers to set research priorities and raise public understanding and acceptability of the use of this data for research. Our workshop provided a useful exercise to identify patient and public views on involvement in big data research within the field of palliative care. These findings provide an initial stepping stone in the path to building guidance for undertaking meaningful involvement in these types of studies, though further consultation with the public and researchers across settings is needed. Continuing to share examples of involvement in big data projects will also be important to increase researchers understanding, and confidence, of involving the public in these projects.
